# Open heart surgery in Nigeria; a work in progress

**DOI:** 10.1186/1749-8090-8-6

**Published:** 2013-01-12

**Authors:** Bode Falase, Michael Sanusi, Adetinuwe Majekodunmi, Barakat Animasahun, Ifeoluwa Ajose, Ariyo Idowu, Adewale Oke

**Affiliations:** 1 Cardiothoracic Division, Lagos State University College of Medicine, Lagos State University Teaching Hospital, Ikeja, Lagos, Nigeria; 2 Department of Anaesthesia, Lagos State University College of Medicine, Lagos State University Teaching Hospital, Ikeja, Lagos, Nigeria; 3 Paediatric Cardiology Unit, Department of Paediatrics, Lagos State University College of Medicine, Lagos State University Teaching Hospital, Ikeja, Lagos, Nigeria; 4 Adult Cardiology Unit, Department of Medicine, Lagos State University College of Medicine, Lagos State University Teaching Hospital, Ikeja, Lagos, Nigeria

**Keywords:** Open heart surgery, Experience, Challenges, Lagos, Nigeria

## Abstract

**Background:**

There has been limited success in establishing Open Heart Surgery programmes in Nigeria despite the high prevalence of structural heart disease and the large number of Nigerian patients that travel abroad for Open Heart Surgery. The challenges and constraints to the development of Open Heart Surgery in Nigeria need to be identified and overcome. The aim of this study is to review the experience with Open Heart Surgery at the Lagos State University Teaching Hospital and highlight the challenges encountered in developing this programme.

**Methods:**

This is a retrospective study of patients that underwent Open Heart Surgery in our institution. The source of data was a prospectively maintained database. Extracted data included patient demographics, indication for surgery, euroscore, cardiopulmonary bypass time, cross clamp time, complications and patient outcome.

**Results:**

51 Open Heart Surgery procedures were done between August 2004 and December 2011. There were 21 males and 30 females. Mean age was 29 ± 15.6 years. The mean euroscore was 3.8 ± 2.1. The procedures done were Mitral Valve Replacement in 15 patients (29.4%), Atrial Septal Defect Repair in 14 patients (27.5%), Ventricular Septal Defect Repair in 8 patients (15.7%), Aortic Valve Replacement in 5 patients (9.8%), excision of Left Atrial Myxoma in 2 patients (3.9%), Coronary Artery Bypass Grafting in 2 patients (3.9%), Bidirectional Glenn Shunts in 2 patients (3.9%), Tetralogy of Fallot repair in 2 patients (3.9%) and Mitral Valve Repair in 1 patient (2%). There were 9 mortalities (17.6%) in this series. Challenges encountered included the low volume of cases done, an unstable working environment, limited number of trained staff, difficulty in obtaining laboratory support, limited financial support and difficulty in moving away from the Cardiac Mission Model.

**Conclusions:**

The Open Heart Surgery program in our institution is still being developed but the identified challenges need to be overcome if this program is to be sustained. Similar challenges will need to be overcome by other cardiac stakeholders if other OHS programs are to be developed and sustained in Nigeria.

## Background

Definitions of Open Heart Surgery vary. For the purposes of this study we defined Open Heart Surgery (OHS) as “surgical repair of the heart during which the blood circulation is often maintained mechanically” [1]. We therefore excluded all the cases done which did not require Cardiopulmonary Bypass (CPB).

There has been limited experience with OHS in West Africa with only a few established cardiac centres [2-4]. Experience is even more limited in Nigeria as most fledgling centres have been unable to overcome the myriad of challenges encountered in establishing OHS in a developing country [2-4] though there is a high prevalence of surgically correctable disease in Nigeria [5-7]. OHS is relatively expensive as income is low in Nigeria, with a recent World Bank report putting the Gross Domestic Product per capita income at just $1,248 as compared to the United Kingdom with a Gross Domestic Product per capita of $35,686 [8]. A small fraction of patients are sponsored or are able to fund their own surgery abroad but the goal for any country has to be to establish its own programs which can be developed and sustained. Despite some early attempts to develop OHS in Nigeria [2,3,9] this has not been sustained. An OHS program is being developed at the Lagos State University Teaching Hospital. The aim of this article is to share the experience in developing this program and discuss the challenges that need to be overcome to further develop and sustain it.

## Methods

### Institutional settings

For several years the Lagos State Government (LSG) has sponsored needy patients to undergo OHS abroad. In 2004 the Global Eagle Foundation (GEF) based in Atlanta expressed an interest in performing OHS in Lagos. The LSG converted a ward into a makeshift cardiac centre which was named the Critical Care Unit (CCU). The CCU consists of a theatre, 4 intensive care beds, 4 side rooms and a side laboratory. A "Cardiac Mission" was then organized with patients recruited and screened by the local cardiologists and surgery performed by the GEF team which also provided all the equipment and materials required for OHS. The first 5 OHS cases were performed in 2004. Further cardiac missions were organized in 2005 and 2006. It became obvious that cardiac missions could not be sustained ad infinitum so there was a drive to develop a local Cardiac Team as well as procure all the necessary equipment for Cardiopulmonary Bypass (CPB) and postoperative monitoring. This was necessary as the equipment donated by GEF rapidly became obsolete and unserviceable. This period of building up the material and human resources occurred in 2007 and 2008 so there was no further surgical activity for those 2 years. By 2009 enough material and human resources had been developed for our institution to recommence the OHS programme. Surgery since 2009 has been performed by both the local team and visiting expert teams.

### Current surgical setup

The core of the local Cardiac Team is made up of 2 Cardiac Surgeons, 3 Cardiologists, 1 Perfusionist, 1 Cardiac Anaesthetist, 1 Intensivist, 1 Cardiac Physiologist and 3 Theatre Nurses. This team works with the CCU which contributes a further 3 Theatre Nurses and 17 Intensive Care Nurses.

A surgical database was designed (Microsoft Access) and has been prospectively maintained since 2006. This is based on the minimum dataset for Cardiac Surgical Activity as proposed by the Society of Cardiothoracic Surgeons of the United Kingdom and Ireland [10]. The database includes the ability to compute risk scores (Parsonnet, Logistic Euroscore and Euroscore) from the inputted data and was the source of the data for this article.

The Cardiac Unit has a dedicated hospital account as well as accounting officers. OHS consumables are sourced from abroad as well as from a developing network of enterprising local medical suppliers. A surgical store is also available for storage of these consumables. During the early cardiac missions, all procedures were entirely paid for by the LSG. Currently, funding has been reduced so patients or their sponsors have to raise the necessary funds for surgery. Following patient referral, clinical assessment and appropriate investigations are done. The euroscore is computed and if deemed acceptable the patient is offered surgery locally. High risk patients are discussed with Cardiac Centres abroad for referral. Patients for local surgery are put on a waiting list till the cost of surgery has been raised. Once a group of 3 or more patients have raised the needed funds, they are batched together for surgery over a number of concurrent days. A decision is made as to whether surgery can be done by the local team or invite a foreign team to assist with surgery. Patients are admitted a few days before surgery and a checklist is used to assess that there are no impediments to the surgery. Following aortic and bicaval cannulation, cardiopulmonary bypass was instituted with the heart lung machine. During the early cardiac missions, the Cobe heart lung machine (Cobe, USA) was in use, but since 2009 all cases have been done with the Stockert Compact heart lung machine (Sorin, Italy). Myocardial protection was with blood cardioplegia in all cases, combining antegrade and retrograde approaches with moderate systemic hypothermia (30-32°C).

Following surgery the patients were transferred to the Intensive Care section of CCU and once weaned off cardiac or respiratory support, the patients were moved to a side room. Total stay in CCU was usually 7–10 days. Patients were discharged home from the CCU, or on occasion spent a few days of further convalescence on the cardiothoracic ward prior to discharge. The patients were usually seen 2 weeks afterwards in the cardiothoracic clinic, and further follow up was done in the cardiology clinic.

Permission was obtained from the Ethics Committee of the Lagos State University Teaching Hospital for use of the existing patient data from the database. The results are presented below, as well as the challenges encountered in achieving these results. Data analysis was done with Microsoft Excel 2010. Data are expressed as absolute values, percentages, or mean ± SD where appropriate.

## Results

**Figure 1:**
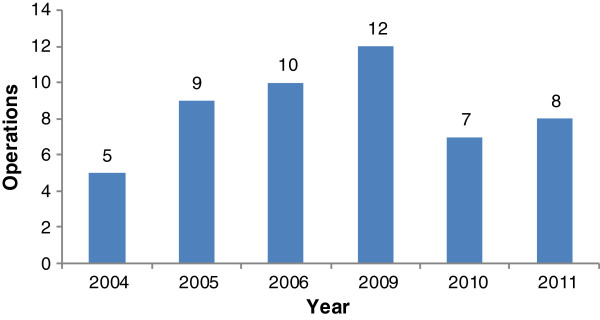
**Annual distribution of OHS procedures.** This graph shows the annual distribution of OHS cases between 2004 and 2011. There was no activity for 2007 and 2008.

**Figure 2:**
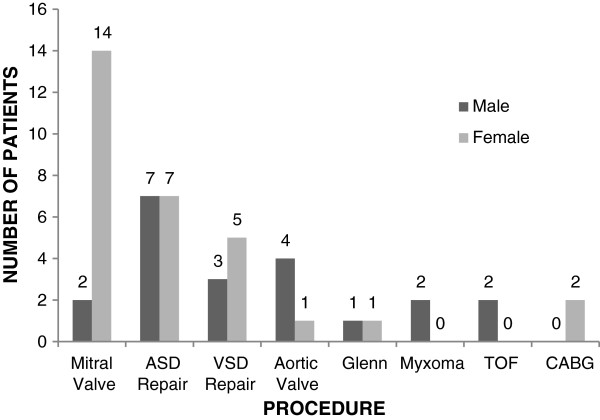
**Sex distribution of OHS patients.** This graph shows the male and female distribution for the different types of OHS procedures.

Between March 2004 and December 2011, 51 OHS cases were performed as seen in Figure 1 which shows the annual distribution of cases. There were 21 males and 30 females with a male to female ratio of 0.7:1. Patient ages ranged from 2 – 72 years with a mean age of 29 ± 15.6 years. The male to female distribution for each procedure is shown in Figure 2. Table 1 shows the various pathologies encountered and types of OHS procedures performed. Table 2 is a summary of the procedures which includes the distribution of OHS procedures, age, euroscore, cardiopulmonary bypass time, cross clamp time and mortality. Table 3 is a summary of the OHS cases done by visiting teams and Table 4 shows cases done by the local cardiac team. Table 5 gives details of the various mortalities.

**Table 1:** Clinical diagnosis, pathology and procedure performed for the OHS cases

**Diagnosis**	**No.**	**Pathology**	**No.**	**Procedure**	**No.**
Atrial Septal Defect	14	Secundum	11	Autologous Pericardium	8
				Direct closure	3
		Primum	2	Autologous Pericardium	2
		Sinus Venosus	1	Haemashield patch	1
Ventricular Septal Defect	8	Perimembranous	8	Dacron patch	7
				Haemashield patch	1
Mitral Valve disease	16*	Stenosis	7	Mechanical Valve	9
		Mixed	7	Biological Valve	5
		Regurgitation	2	CE Annuloplasty Ring	1
Aortic Valve disease	5	Regurgitation	5	Mechanical Valve	5
Myxoma	2	Left Atrium	2	Excised with septal button	2
				Autologous pericardial	
				Patch closure	
Tetralogy of Fallot	2			RVOT transannular patch	2
				Ventricular muscular band excised.	
				Pulmonary valve excised (1)	
				Pulmonary valve spared (1)	
Hypoplastic Right Ventricle	2	Tricuspid atresia	2	Bidirectional Glenn Shunt	2
Ischaemic Heart Disease	2			CABG x 2 (RSVG to Cx, RCA)	1
				OPCAB x 1 (LIMA to LAD)	1
TOTAL	51				50*

* . A patient intended for MVR did not actually have a valve implanted as surgery was aborted (patient number 4 in Table 5).

*RSVG* Reversed Saphenous Vein Graft, *OPCAB* Off Pump Coronary Artery Bypass Grafting, *LIMA* Left Internal Mammary Artery, *Cx* Circumflex Coronary Artery, *RCA* Right Coronary Artery, *LAD* Left Anterior Descending Coronary Artery, *RVOT* Right Ventricular Outflow Tract, *CE* Carpentier Edwards.

**Table 2:** Age, euroscore, operative details and mortality for OHS categories

**Operation**	**No (%)**	**Age (years)**	**Euroscore**	**CPB (mins)**	**XClamp (mins)**	**Mortality**
MVR	15 (29.4%)	33.2 ± 11.4	3.9 ± 1.3	145.5 ± 41.1	96.3 ± 20.6	2
MV Rep	1 (2%)	21	3	120	80	0
ASD	14 (27.5%)	28.9 ± 12.4	2.9 ± 0.9	86.5 ± 44.2	45.9 ± 29.8	1
VSD	8 (15.7%)	15.0 ± 9.4	3.9 ± 1.2	159.1± 77.3	108.9 ± 64.7	2
AVR	5 (9.8%)	23.8 ± 3.1	4.0 ± 1.4	156.8 ± 10.8	103.5 ± 20	0
Glenn	2 (3.9%)	13 ± 2.8	2.5 ± 0.7	97 ± 1.4	N/A	0
LA Myxoma	2 (3.9%)	60 ± 14.1	7 ± 4.3	132 ± 11.3	98 ± 2.8	1
TOF	2 (3.9%)	15.5 ± 13.4	2	267.5 ± 19.1	127 ± 15.6	2
CABG	2 (3.9%)	64 ± 11.3	10 ± 5.7	160	70	1
**TOTAL**	**51**					**9**

*MVR*: Mitral Valve Replacement, *MV Rep* Mitral Valve Repair, *ASD* Atrial Septal Defect, *VSD* Ventricular Septal Defect, *AVR* Aortic Valve Replacement, *Glenn* Bidirectional Glenn Shunt, *LA Myxoma*, Left Atrial Myxoma, *TOF* Tetralogy of Fallot, *CABG* Coronary Artery Bypass Grafting, *CPB* Cardiopulmonary Bypass time, *Xclamp* Aortic Cross Clamp time.

**Table 3:** Cardiac missions, visiting team, OHS procedures performed and mortalities

**Serial**	**Team**	**Period**	**OHS procedure**	Mortality (*)
1	GEF	January 2004	ASD-2	MVR(*1)
			VSD-1	
			AVR-1	
			MVR-1	
2	GEF	June 2005	ASD-2	
			VSD-1	VSD(*2)
			AVR-3	
			MVR-3	
3	GEF	March 2006	ASD-3	None
			VSD-2	
			MVR-4	
			LA Myxoma-1	
4	GEF	Jan-Feb 2009	ASD-2	ASD(*3)
			VSD-1	
			AVR-1	
			MVR-3	MVR(*4)
5	ARI	April 2009	ASD-1	None
			VSD-1	
			MVR-1	
6	GEF	February 2010	CABG-1	CABG(*5)
7	RH	June 2011	ASD-1	
			VSD-2	VSD(*6)
8	FORTIS	November 2011	MVR-1	None
			Mitral Valve Repair-1	
			OPCAB-1	
	TOTAL		42	6

(*) Mortality details in Table 5.

*GEF* Global Eagle Foundation Atlanta USA, *ARI* Aberdeen Royal Infirmary Scotland, *RH* Ruby Hall Clinic Pune India, *FORTIS* Fortis Hospital, Bangalore India.

**Table 4:** Periods of operating, procedures performed and mortality of OHS Cases done by the local cardiac team

**Serial**	**Period**	**OHS procedure**	Mortality (*)
1	April 2009	MVR-1	None
2	January 2010	ASD-1	None
3	May 2010	Glenn-1	None
4	October 2010	ASD-1	TOF Repair(*7)
		Glenn-1	
		TOF Repair-1	
5	December 2010	TOF Repair-1	TOF Repair(*8)
6	May 2011	LA Myxoma-1	LA Myxoma(*9)
7	December 2011	ASD-1	None
	**TOTAL**	**9**	**3**

(*) Mortality details in Table 5.

**Table 5:** Age, procedure, timing of procedure, euroscore and cause of death

No (*)	**Age**	**Procedure**	**Timing**	**Euroscore**	**Cause of death**
1	17	MVR	Elective	7	Severe Pulmonary Hypertension. Unable to wean off CPB.
2	10	VSD	Elective	3	PDA missed preoperatively. Required circulatory arrest. Failed to come off CPB
3	25	ASD	Elective	4	Post op SVT, sudden cardiac arrest.
4	21	MVR	Elective	7	Myocardial ischaemia due to failure to achieve cardioplegic arrest following aortic cross clamping
5	72	CABG	Urgent	14	Recent MI. RV aneurysm. Preop IABP. Progressive Post op RV failure
6	2	VSD	Elective	4	Progressive post op RV failure
7	6	TOF	Elective	2	Progressive post op RV failure
8	25	TOF	Elective	2	Severe pyrogenic blood transfusion reaction, renal failure. Hepatitis B positive. Turned down for dialysis.
9	70	LA Myxoma	Urgent	10	Cardiogenic shock prior to surgery. Failed to come off CPB

(*) Mortality sequence from Tables 3 and 4.

*MI* Myocardial Infarction, *RV* Right Ventricle, *SVT* Supraventricular tachycardia, *IABP* Intra-aortic balloon pumping.

The complications seen in this case series were inotropic support in 19 patients (37.3%), atrial fibrillation/supraventricular tachycardia in 6 patients (11.8%), ventricular fibrillation in 3 patients (5.9%), chest infection requiring antibiotic treatment in 7 patients (13.7%), pleural effusion requiring drainage in 6 patients (11.8%), superficial sternal wound infection in 4 patients (7.8%), re-operation for bleeding in 2 patients (3.9%), acute renal failure in 3 patients (5.9%), pericardial effusion in 1 patient (2%) and transient stroke in 1 patient (2%). The overall mortality in the series was 9 patients (17.6%) and the mean euroscore for the mortalities was 5.8 ± 4.3.

## Discussion

The 30 day mortality rate in this series of 17.6% is high by western standards but is not dissimilar to that of established OHS programs in West Africa where 30 day mortality from published data ranges from 5-13% [11,12]. These mortalities need to be viewed in the context of the particular challenges presented by our environment. These challenges will be highlighted as the mortalities are discussed.

Limitations in the diagnostic armamentarium in some cases led to missed diagnosis and in others failure to adequately assess the risk for surgery. Patient 1 had severe pulmonary hypertension as the pulmonary artery was found to be twice the size of the aorta intra operatively (Table 3). Cardiac catheterization was not available at this stage and the echocardiography machine was of limited functionality with poor resolution. This also contributed to the problems in patient 2 who underwent repair of a Ventricular Septal Defect (VSD) but a Patent Ductus Arteriosus (PDA) was missed during preoperative transthoracic echocardiogram (Table 3). Following institution of CPB, aortic cross clamping, cardioplegic arrest of the heart and opening of the right atrium, the Perimembranous VSD could not be visualized due to copious return of aortic blood to the right ventricle. A PDA was suspected but despite reducing CPB flows to reduce aortic return and improve visualization, it could not be located. Following cooling, circulatory arrest was instituted and the PDA was successfully located and ligated. The VSD was then repaired. Though the patient came off CPB he deteriorated postoperatively and died.

Though attempts were made to stick to simple cases in the early years as has been recommended for fledgling programmes [13,14] this was not always possible as if cases are turned away they often have no other recourse to surgery. This was the case with patients 5 and 9 despite euroscores of 14 and 10 respectively (Table 3). Both patients presented in cardiogenic shock, patient 5 following dissection of the circumflex coronary artery during percutaneous transluminal angioplasty and patient 9 secondary to obstruction of the mitral valve from a giant left Atrial Myxoma.

Inadequate stocks of consumables as well as missed preoperative diagnosis were contributory to the mortality in patient 4 (Table 3). She was scheduled for Mitral Valve replacement but moderate aortic regurgitation which was missed preoperatively meant that antegrade cardioplegia regurgitated into the left ventricle with ensuing distension. Retrograde cardioplegia was also unsuccessful as the retrograde catheter would not stay in the aneurysmal coronary sinus. Cannulae for direct coronary ostial cannulation were not available and the procedure was therefore abandoned. The patient however required progressively increasing inotropic support and eventually died of myocardial failure 24 hours post operatively.

The first Tetralogy of Fallot (TOF) case (patient 7) succumbed to right ventricular failure following placement of a transannular pericardial patch in the right ventricular outflow tract and resection of the pulmonary valve leaflets as the right ventricle appeared to be unable to tolerate the ensuing pulmonary regurgitation. In the second TOF case (patient 8) we were able to spare the valve and avoid pulmonary regurgitation. The patient did very well initially postoperatively, being extubated on minimal inotropic support. At the time the hospital blood bank had limited blood supplies and packed cells required for transfusion was obtained from a private blood bank. This resulted in a severe pyrogenic blood transfusion reaction with severe hypotension which though controlled, resulted in acute renal failure. The patient was subsequently refused renal dialysis as he was hepatitis B positive and the hospital had just two dialysis machines (and there were concerns that Hepatitis B would be spread to other patients). Prior to completing arrangements for alternate dialysis he deteriorated further and died.

Of the 42 OHS cases done by visiting teams, there were 6 mortalities (Table 3). Out of the 9 cases done by the local team there were 3 mortalities (Table 4). Overall mortality was therefore 17.6%. Table 5 summarises the cause of death for each mortality.

A number of lessons have been learnt from the challenges faced that resulted in mortalities. The equipment and consumable base has been strengthened as a cardiac store has been established and links developed with various medical suppliers. All patients are screened preoperatively and without exception are excluded if they are positive for HIV or Hepatitis. More care is being taken to rigidly exclude high risk cases and emergency cases unfortunately can’t be done at this stage of our development. Blood bank facilities have been improved and in addition time is spent recruiting multiple donors to get freshly screened and cross matched blood for surgery. Blood from outside private laboratories is no longer allowed.

### Challenges

The results described above have been achieved against a background of a number of challenges. These challenges are discussed below. Though discussed separately for ease of discussion there is considerable overlap between these challenges.

1. **Low volume of cases.** Only 51 cases have been performed over the 6 years of active surgery, averaging about 8 cases annually. Most of these cases have been done in batches, so there are periods of activity in between which there is no OHS activity. Apart from the cases in the early years which were funded entirely be LSG for Cardiac Missions, funds now need to be sought for referred patients which is not always successful, and causes long delays. Some cases are assessed as being too challenging to be done locally with the current constraints in the working environment (discussed below) and are referred abroad. Most patients that attend our clinics are self-referred or from our institution. Referrals from Cardiologists outside our institution are almost non-existent. It is unfortunately more profitable for most cardiologists to refer patients to facilities abroad as a stipend is received for such referrals. This medical tourism impacts negatively on local development of OHS programmes and contributes to the low number of cases done. This causes a vicious cycle as with the low volumes it has been difficult to generate the positive results that would build up the confidence of the public and referring cardiologists to encourage more referrals.

2. **Unstable working environment.** The model for OHS in our institution has involved the surgery being done in CCU. This has raised a number of challenges which are not unusual in a developing country. Funding is irregular which has caused difficulty in maintaining equipment and stock of consumables. Like the rest of the country, electrical power is a major issue as the supply from the national grid is unreliable and the power surges which often occur risk damage to vital equipment. The hospital is powered by 5 generators, 2 of which are connected to the CCU. As these are in constant use they often break down and it is therefore part of the checklist to liaise with the engineering department to be sure power is guaranteed during the period of surgery and immediate recovery. Postoperatively the power issue has been partly addressed by an inverter system which powers two of the beds in the intensive care unit and ensures that critical equipment have backup power for at least 6–8 hrs. In addition, all equipment purchased has the backup of internal batteries.

3. **Training and exposure.** Some members of the cardiac team have had foreign training and exposure while other members of the team have had more limited exposure to OHS. The low volume of cases as discussed above has severely limited the hands-on exposure available and is also leading to deskilling of the trained staff. As suggested by Ghosh [13] the minimum number of OHS cases annually to provide training and maintain skills should be 100. Our average of 8 cases annually falls well short of this. Some members of the team have been sent for short periods of exposure in high volume centers in India. Further funding is required to be able to sustain these efforts. In addition regular surgery needs to become the norm otherwise these personnel return but are unable to apply the skills they have acquired and end up losing these skills. Visits by foreign expert teams have been too brief and infrequent to allow for regular skills transfer. Efforts have been made to get foreign teams to visit more frequently but unfortunately financial considerations have so far limited this option.

4. **Laboratory support facilities.** Successful OHS requires 24 hour laboratory support, an active blood bank and cardiac catheterization support. Access to these various support facilities has been very variable and limited over the years though this is gradually being developed. Support is available during working hours from the hospital laboratories for full blood counts, clotting profiles, electrolytes and liver function tests. Arterial blood gases were initially available in the side laboratory in CCU but as CCU funding dried up the Cardiac Unit had to source for alternatives. Arterial blood gases are now done using a point of care device called the I-stat (Abbott, England) which also covers basic electrolytes. Activated clotting times as well as prothrombin time/International Normalized Ratio are done using a Hemochron Junior.

#### Radiology

This has been particularly challenging. In the early cardiac missions transthoracic echocardiography was available using a portable ultrasound machine (sonosite) which lacked adequate resolution and contributed to some cases being wrongly or inadequately diagnosed. This was improved with the purchase of a Vivid I echocardiogram machine (GE Healthcare Systems England) which has enhanced echocardiographic diagnosis as well as made available transoesophageal echocardiography. Chest radiography is available at the hospital radiology department but despite the purchase of a portable X-ray machine great difficulty is experienced in trying to get portable chest radiograms done, largely due to local logistical issues. The majority of OHS cases done therefore do not get portable chest radiograms.

#### Blood bank facilities

The haematology department has been very supportive but has a number of constraints. Blood products can be obtained (fresh frozen plasma, platelets, cryoprecipitate) but require a lot of advance notice for preparation. Preparation of the products is not always possible onsite (erratic electrical power supply, equipment failure) and is sometimes ordered and prepared at the blood bank of a regional general hospital about 20 kilometers away. Blood donors are not readily available as there are local concerns among the populace about having their blood screened for HIV. Relations and friends of intending patients are therefore encouraged to donate. Routine preparation for each OHS case that will require CPB is 4 units of packed cells, 4 units of Platelets, 4 units of Fresh Frozen Plasma and 1 unit of Cryoprecipitate. A blood fridge is available in CCU for storage of the blood products. As further blood products can rarely be obtained if needed urgently for postoperative bleeding strenuous efforts are made to minimize the risk of bleeding. Antifibrinolytics are used routinely (initially Trasylol now Tranexemic Acid). Case selection is limited to those where relatively short pump runs can be achieved. Care is taken to limit the priming volume and ultrafiltration is used where necessary. The screening of blood in private blood banks can be lax with resultant complications as seen in the patient who underwent successful repair of TOF but died from complications of blood transfusion (patient 8). Blood is therefore no longer obtained from private blood banks.

#### Cardiac catheterization

Cardiac catheterization has only become available since 2009. It is currently provided by a large private hospital in Lagos and costs approximately 2000 US Dollars. This is considerably higher than what obtains in countries like India where it can cost as low as 200 US Dollars. Till more centres open and the cost is lower it will limit the availability of this vital diagnostic tool. The lack of cardiac catheterization also limited patient selection to largely those less than 40 years of age who were unlikely to have ischaemic heart disease. This accounts for the mean age in this series of 29 ± 15.6 years.

5. **Financial support.** In a separate study we have determined the cost of various OHS procedures in our programme. Surgery for ASD closure costs $6,230, Coronary artery bypass grafting $8,430 and Mitral valve replacement $11,200 (personal data). The per capita income in Nigeria is however only 2 USD daily! This therefore makes it very difficult for patients to afford OHS. This has been the Achilles’ heel of our programme since its inception and has severely limited the number of operations that can be done. Urgent assistance in seeking funding for patients is therefore required and the options available include the government, non-governmental organizations, foundations and establishment of national health insurance schemes [11,13,14].

6. **Moving away from the “Cardiac Mission” Model.** The experience at our Institution highlights the huge difficulty in a developing country of making the transition from the model of intermittent cardiac missions to what obtains in the western world and a few cardiac centres in Africa of regular sustainable surgery. The cardiac mission model is not a sustainable one as a lot of effort and expenditure is allocated towards surgery on a few patients [13-15]. These missions are led by foreign experts who can only afford short periods away from their regular jobs. Incurred expenditure may go towards paying for travel of the foreign team, visas, air freight of needed equipment and consumables, local accommodation and transportation. In some cases these experts request exorbitant professional fees for their services. The visiting team has limited time for the exercise which limits the number of cases that can be done. Efforts are made to avoid high risk cases which would consume scarce resources, operating time and tie down intensive care beds [13]. In addition there is the higher risk of mortality if high risk cases are taken on with attendant negative publicity and loss of morale of the cardiac team. Financial support for cardiac missions is very variable as it depends on both political expediency and the priorities of the Government of the day.

Our institution has had some limited success in moving away from the model of cardiac missions but completing the transition from a model of cardiac missions to one of an indigenous programme with regular funding for OHS activity has been very challenging and has resulted in the OHS program in our institution remaining in its infancy as alluded to by Nwiloh et al. [15]. It has often been easier to get funding for cardiac missions than for surgery by the local team. The added burden of the challenges discussed above has severely limited the number of cases that could be done, especially the challenge of an increasingly unstable working environment. Of the 51 OHS cases only 9 have been performed by the local team. All the challenges earlier mentioned need to be addressed to achieve the goal of an independent indigenous programme.

## Conclusions

The challenges we face are not unique to our institution. These same challenges have hindered the development of other OHS programs in Nigeria as well as in the West African sub-region. Nigeria, Ghana, Cote D' Ivoire, Senegal, Cameroun and Mauritania have all at various times had OHS activity. Of these, only Cote D'Ivoire and Ghana have been successful in maintaining and sustaining active programs with regular OHS activity [11,16]. The challenge of Open Heart Surgery in Nigeria was recently highlighted by Nwiloh et al. who described their experience with OHS in Northern Nigeria [15]. The challenges included lack of equipment and ancillary support services as well as lack of trained personnel. The 2 deaths in their series of 18 patients were ascribed to the lack of blood bank capabilities to provide component blood therapy and the unavailability of potent broad spectrum antibiotics. These are problems that would be inconceivable in OHS programs in the developed world but we have to grapple and contend with in programs in the developing world. An understanding of the challenges faced in West Africa is not complete without referring to the recent monograph by Professor Adebonojo who asks "Why Did Heart Surgery Programmes in Nigeria Fail?" [17]. He attributes the failure to the heavy financial outlay, intensive labour and high resource consumption of performing OHS. The depressed economy, wastage of scarce resources and fragile and unstable governments are also contributory factors. Disorganized planning, decentralization of efforts, lack of national health statistics, national health insurance scheme, national planning, medical, surgical and nursing manpower further compound the challenge [3,4,17].

It is important to note that the LSG is approaching completion of the construction of a new stand-alone cardiac facility. This incorporates a dedicated theatre, a cardiac catheterization suite, 5 Intensive care beds, 7 high dependency beds and 20 ward beds. It has clinic facilities, side laboratories for blood investigations, radiology facilities (X-ray, ultrasound, CT scan) and a pharmacy. This centre is due to become operational later this year. The organizational and management structure as well as funding are yet to be determined. The past holds the key to the future. The experience gained from our OHS program to date highlights the challenges which if addressed will help ensure this new facility becomes a robust and vibrant OHS program.

It is hoped that the experience at our institution as described in this article will offer useful pointers to other cardiac stakeholders in Nigeria and allow them to identify the potential landmines as they look to starting new programs or resuscitate flagging programs.

We are indebted to the efforts of the early pioneers of OHS in Nigeria [2,3,9]. Without their courage and commitment we could never have come this far. The African continent [18] and Nigeria is facing a rising epidemic of cardiac disease [19-24]. This mandates urgently addressing and overcoming the challenges to developing OHS in Nigeria. The development of Open Heart Surgery in Nigeria is however now at a crossroads. It is a daunting task to take on with all the challenges we and others have highlighted. A flame has however begun to burn in our institution and could be a beacon to others to join hands with us to nurture and sustain it. We have only two ways to go, up or down. The biggest challenge to our success has always been the financial support for OHS. The Government is unlikely to be able to solely carry the burden for OHS as there are numerous competing social priorities. We therefore call on heart foundations and non-governmental organizations to assist us in moving OHS forward in our institution and in Nigeria.

## Abbreviations

OHS: Open Heart Surgery; LSG: Lagos State Government; GEF: Global Eagle Foundation; CCU: Critical Care Unit; CPB: Cardiopulmonary Bypass; VSD: Ventricular Septal Defect; PDA: Patent Ductus Arteriosus; TOF: Tetralogy of Fallot; CE: Carpentier Edwards; RVOT: Right Ventricular Outflow Tract; CABG: Coronary Artery Bypass Grafting; RSVG: Reversed Saphenous Vein Graft; Cx: Circumflex Coronary Artery; RCA: Right Coronary Artery; OPCAB: Off Pump Coronary Artery Bypass Grafting; LIMA: Left Internal Mammary Artery; LAD: Left Anterior Descending Coronary Artery; MVR: Mitral Valve Replacement; MV Rep: Mitral Valve Repair; ASD: Atrial Septal Defect; AVR: Aortic Valve Replacment; Glenn: Bidirectional Glenn Shunt; LA: Left Atrial; RH: Ruby Hall Clinic Pune India; ARI: Aberdeen Royal Infirmary; FORTIS: Fortis Hospital Bangalore India; SVT: Supraventricular Tachycardia; MI: Myocardial Infarction; RV: Right Ventricle; IABP: Intra-aortic Balloon Pumping.

## Competing interests

The Authors declare that they have no competing interests.

## Authors’ contributions

BF conceived of the study, extracted the patient data, did the data analysis, wrote the first draft, and reviewed the second draft. MS reviewed the first draft and reviewed the second draft. AM reviewed the second draft and helped with the literature search. AA made intellectual contributions to the second draft and reviewed the second draft. IA assisted with the extraction of patient data, assisted with the data analysis and reviewed the second draft. AI made intellectual contributions to the second draft and reviewed the second draft. AO reviewed the final draft and made intellectual contributions. All the authors approved the final draft for submission.
